# Balance Improvement and Fall Risk Reduction in Stroke Survivors After Treatment With a Wearable Home-Use Gait Device: Single-Arm Longitudinal Study With 1-Year Follow-Up

**DOI:** 10.2196/67297

**Published:** 2025-08-15

**Authors:** Brianne Darcy, Lauren Rashford, David Huizenga, Kyle B Reed, Stacy J M Bamberg

**Affiliations:** 1 Moterum Technologies, Inc., 2757 S 300 W Ste F, Salt Lake City, UT, 84115, United States, 1 315-335-9622; 2 Department of Mechanical Engineering, University of South Florida, Tampa, FL, United States

**Keywords:** stroke, falls, balance, gait device

## Abstract

**Background:**

Falls are a common and serious problem after stroke, often leading to injuries, loss of independence, and increased health care usage. Functional balance, a primary risk factor for falls, is frequently impaired in individuals with hemiparetic gait impairments. Previous research with the iStride gait device (Moterum Technologies, Inc) showed that functional balance improved immediately following 4 weeks of treatment. However, the long-term retention of these effects remains unknown and could improve the management of balance and mobility impairments after stroke.

**Objective:**

This study aimed to determine the long-term functional balance effects of treatment with the gait device for individuals with hemiparetic gait impairments from stroke.

**Methods:**

Eighteen individuals with chronic stroke (9 male, 9 female, mean age 57 years, and 60 months post stroke) participated in twelve 30-minute treatment sessions with the gait device. During each treatment session, the device was worn on the less affected lower extremity during overground ambulation in the participant’s home. All treatment and assessments were overseen by licensed physical therapists. Functional balance was evaluated using the Berg Balance Scale (BBS), the Timed Up and Go (TUG) test, and the Functional Gait Assessment (FGA) at baseline and 5 posttreatment follow-ups: 1 week, 1 month, 3 months, 6 months, and 12 months after treatment. Balance improvement was analyzed using repeated-measures ANOVA from baseline to each follow-up time frame, correlation analysis, comparison to each outcome’s minimal detectable change (MDC) value, evaluation of fall risk classification changes, and subjective questionnaires.

**Results:**

Participants retained statistically significant improvements on the BBS, TUG, and FGA compared with baseline at all posttreatment time frames (*P*<.05). All participants initially identified as being at risk for falls reduced their fall risk on at least one outcome during one or more follow-up assessments. At 12 months post treatment, the average improvement on all 3 outcomes remained above their respective MDC thresholds, demonstrated by a 5.9-point improvement on the BBS, a 4.9-second improvement on the TUG, and a 34.6% (3.8-point) improvement on the FGA. At least 72% of participants exceeded the MDC of BBS, at least 44% exceeded the MDC of TUG, and at least 66% exceeded the MDC of FGA at every posttreatment time point. Subjective questionnaire responses indicated that 88% of participants perceived functional balance improvement following treatment with the gait device.

**Conclusions:**

The findings of this study indicate that treatment with the gait device may result in long-term functional balance improvement for individuals with hemiparetic gait impairments from stroke. Larger, controlled studies are recommended to confirm these findings.

## Introduction

Falls are a common and serious problem after stroke. In the first year following a stroke, approximately 70% of individuals fall [1-3], and the risk of falls remains substantially elevated throughout the stroke survivor’s lifespan [2]. The physical consequences of falls can be devastating, often leading to hospitalizations, serious injuries, and even death [4]. Stroke survivors who fall are also especially vulnerable to adverse outcomes from falls, such as a 4-fold increase in fracture risk compared with healthy controls [5], and more severe fracture consequences [2].

In addition to physical complications, the psychological consequences of falls after stroke can be severe. Compromised mobility confidence and fear of falling are experienced by up to 88% of stroke survivors who have fallen, and 44% report further restricting their mobility activities after a fall [2]. Given the documented low activity levels of stroke survivors in general [6], further activity restriction reinforces limitations with functional independence. Furthermore, the psychological consequences of falls are associated with depression [3,7], reduced community participation [8], social deprivation [2], and decreased quality of life [4,9], all of which can be detrimental to a stroke survivor’s well-being.

For individuals with stroke, gait and balance impairments are widely regarded as primary fall risk factors [2,10]. Impairments to muscle strength, sensation, proprioception, vision, cognition, and attention [2,11] can further compound this risk and present additional barriers to normalizing mobility and fall risk. As primary risk factors, balance and gait-focused activities are commonly prescribed treatments to reduce fall risk post stroke. However, there is a dearth of high-quality evidence to support the efficacy of specific balance and gait-focused activities [12-14]. As improved medical management continues to extend the life expectancy of stroke survivors [15], successful treatments for balance improvement and fall risk management are essential to improve the health and safety of these individuals.

**Figure 1.**
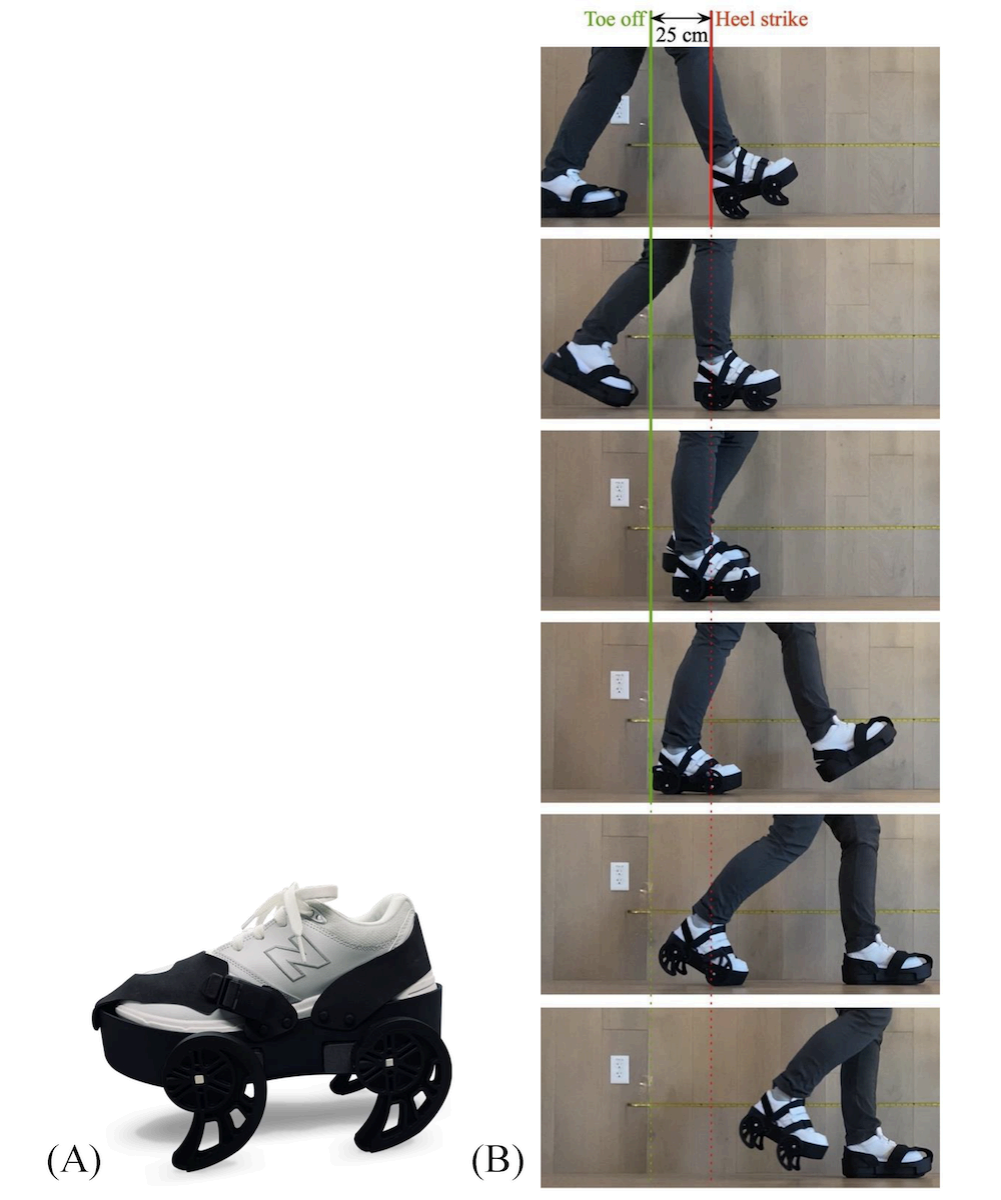
The iStride gait device and mechanism of action. (**A**) The iStride gait device. (**B**) The gait device motion. As the user bears weight into the device during ambulation, the device’s rotating wheels push the nonparetic foot backward during stance. This backward motion promotes usage of the paretic leg by slightly destabilizing the nonparetic leg. To adjust for the added height of the device, a similar height but stationary platform is worn on the foot of the paretic limb.

The iStride gait device [16] (Moterum Technologies, Inc.) was developed to support the rehabilitation needs of the ambulatory stroke population. Specifically, the device targets the asymmetric movement patterns of individuals experiencing hemiparesis, a notable contributor to gait and balance impairments in neurologic populations [17,18]. The device is worn over the shoe on the foot of the nonparetic limb during ambulation. Four rotating, kinetic wheels [19] on the device generate a steady backward translation of this limb during weight bearing in the stance phase of gait. Given the typical reliance or “favoring” of the nonparetic limb by individuals with hemiparesis during ambulation [20], this stance phase motion induces a relative instability, prompting a subtle weight transfer to the affected limb. Resembling principles of lower extremity constraint-induced movement therapy [21], this weight transfer is designed to increase usage of the paretic limb during the gait cycle. In addition, the backward motion of the wheels increases the stride length of the affected limb. These exaggerated mechanics trigger the user to correct this pattern (similar to the aftereffect experienced with split-belt treadmill training [22]) once the device is removed and the individual continues overground ambulation without the device. Further details of the device’s development and mechanisms of action are explained in previously published studies [19,23-25]. Figure 1 shows the gait device and its motion during the gait cycle.

The documented effects of treatment with the gait device include improvements to gait symmetry, specifically step length symmetry and double support symmetry [24,26]. Gait speed has also been shown to increase immediately posttreatment [24,27,28] with effects largely unchanged one year after treatment completion [29]. Additional benefits have been noted in the realm of functional balance. Specifically, a study conducted with participants using the gait device in their home environment found statistically significant improvements on balance-focused functional outcomes following 12 treatment sessions with the gait device. Moreover, fall risk reduction was experienced by 80% of participants, as determined by comparison to fall risk thresholds on one or more outcome measures [27]. While these posttreatment outcomes are promising for an immediate benefit of gait device treatment, retention of these improvements remains unknown and could provide further guidance on the long-term utility of this treatment and its role in managing poststroke balance and mobility impairments. Therefore, the objective of this study was to investigate the long-term effects of gait device treatment on functional balance and risk for falls for individuals with hemiparesis caused by stroke.

## Methods

### Participant Recruitment

Community-dwelling individuals with chronic stroke were recruited to participate in the study using convenience sampling. Inclusion criteria specified: (1) age of 21‐80 years, (2) one or more cerebral strokes (all on the same side), (3) stroke occurred at least 6 months previously, (4) gait asymmetry but can walk independently with or without a cane, (5) no evidence of severe cognitive impairment that would interfere with understanding instructions, (6) not currently receiving physical therapy, (7) no evidence of 1-sided neglect affecting ambulation, (8) adequate walking space within the home, and (9) body weight less than 250 pounds. Individuals were excluded from the study if they had: (1) uncontrolled seizures, (2) pregnancy, (3) metal implants (stents), (4) chronic obstructive pulmonary disease, (5) uncontrolled high blood pressure, (6) myocardial infarction within the last 180 days, (7) head injury within the last 180 days, or (8) a history of a neurologic disorder other than stroke.

### Ethical Considerations

The study was confirmed to meet ethical standards for research with human participants by the Western institutional review board (IRB), now named Western-Copernicus Group IRB. Participants signed a consent form that was approved by Western IRB before participating in the study. Data were coded without personally identifying information and stored using HIPAA (Health Insurance Portability and Accountability Act) compliant software. Compensation for participation in study activities included US $6 for each study visit, and US $50, US $75, and US $250 for follow-up assessment sessions after the treatment period.

### Experimental Setup

The study followed a single group, a before-after design, with multiple follow-ups after the treatment period. Determination of eligibility included an initial phone screen by the principal investigator, followed by a home visit to verify adherence with eligibility criteria and to assess that the home environment met the spatial needs for gait device treatment (at least 25 feet of walking space).

Each participant’s functional balance was measured at baseline (approximately 1 week before starting device treatment). After baseline assessments, the participants completed approximately 4 weeks of gait device treatment followed by 5 follow-up balance assessments: 1 week, 1 month, 3 months, 6 months, and 12 months after treatment. At the 12-month follow-up session, participants were provided a questionnaire regarding their clinical trial experience, fall history before and after treatment with the gait device, and subjectively observed balance changes. All aspects of this study were performed in each participant’s home environment and were overseen by licensed, nonemployee physical therapists hired as contractors for clinical trial data collection. No other treatment or physical therapy services were provided to the participants as a part of this study during or after the treatment period.

### Balance Assessments

Various outcome measures have been validated to assess unique aspects of functional balance [30], and using several balance-focused outcomes is commonly engaged in research and clinical practice to assess balance ability comprehensively [30,31]. The Berg Balance Scale (BBS), the Timed Up and Go (TUG) test, and the Functional Gait Assessment (FGA) were selected in the present study due to their high clinical usage [32,33], validation and recommended usage in the chronic stroke population [34,35], and ability to be performed in the home environment. In addition, each of these outcome measures provides a clinically relevant threshold value to differentiate high and low risk of falls, an objective of this study, as well as criteria depicting the necessary magnitude of improvement to exceed measurement error (ie, the minimal detectable change [MDC]). Each of these 3 outcomes has also been identified as having high-quality (Level 1) evidence to support usage in neurologic populations, and the BBS and FGA are recommended as core outcomes in clinical practice guidelines published by the Academy of Neurologic Physical Therapy [35].

The BBS [36] is a 14-item measure that assesses static balance, dynamic balance, and fall risk. This commonly used and widely studied outcome has various published fall risk and MDC threshold values. For our study, we selected a fall risk threshold value of 44 based on a 2011 study by Simpson et al [37], which studied a similar population of ambulatory, community-dwelling individuals with stroke. Similarly, an MDC score of 2.5 points [38] was selected for our comparison from several alternative thresholds [34,39,40] as their sample provides the most comparable “time since stroke” of approximately 46 months.

The TUG test [41] assesses dynamic balance and walking ability, and this outcome measure has been shown to differentiate individuals with stroke from healthy individuals [42]. A score above 14 seconds is used for predicting falls after stroke [43], and a threshold MDC value of −3.2 seconds indicates a change beyond measurement error for individuals with chronic stroke [34].

The FGA is used to assess postural stability during walking [44]. The FGA does not have a specific fall risk cutoff for the chronic stroke population. Therefore, we selected a related threshold of 15 points [45], used for individuals with Parkinson disease, another neurologic disorder affecting gait and balance. A threshold value of 14.1% or 4.2 points, determined from a sample of individuals with stroke [46], is available for MDC comparison.

### Treatment Sessions

Following the baseline balance assessment, participants underwent treatment with the gait device 3 times per week over 4 weeks, totaling 12 treatment sessions. During each treatment session, the device was worn on the participant’s nonparetic foot, and an approximately height-matched shoe was worn on the paretic foot. Participants walked on the gait device for a target of 30 minutes during each session with rest breaks at scheduled 5-minute intervals, or more frequently if needed. Licensed physical therapists supervised all sessions and provided the level of mobility assistance needed for participant safety and comfort while walking on the gait device.

### Statistical Methods

The sample size for this long-term follow-up study is based on the number of individuals who participated in the 4-week treatment and who continued in the study for the year following treatment. The sample size for the short-term study [27] was derived using power analyses from 2 previous studies with the gait device [23,24]. In the first study [23], the analysis focused on the comparison between pretreatment and posttreatment data in healthy individuals without mobility impairments. It revealed an effect size of 0.68 for the difference in step length asymmetry. These calculations estimated that a sample size of 18 participants would obtain a power of 0.8. The second study [24], based on a pilot in-clinic study using the device with individuals with stroke, calculated an effect size of 0.71 for gait speed. A power analysis based on gait speed showed that 21 participants would obtain a power of 0.85. In the present study, 21 participants completed the treatment. After the treatment period, 3 participants did not complete all remaining follow-up sessions. Since we are reporting repeated-measures statistical tests, this study reports results from the 18 participants who completed all outcome assessments at all time periods.

Statistical tests included both parametric (repeated-measures ANOVA) and nonparametric (Friedman) tests to determine the statistical significance of outcome measure score changes between baseline and each of the 5 follow-up time frames. The Friedman test was used when the data violated the assumptions of an ANOVA test. When statistical significance was found, post hoc pairwise comparisons were performed using Tukey honestly significant difference or the Wilcoxon signed-rank test for nonparametric (Friedman) tests. The Spearman rank-order correlation coefficient was used for correlation analysis. All statistical analyses were calculated using SPSS Version 26 software (IBM Corp).

To evaluate the risk for falls, we compared each participant’s outcome measure score at each time frame to the related fall risk threshold of each outcome measure. In addition, to assess the magnitude of outcome measure change compared with the measurement error of each test, we compared both our study group average and each individual’s score changes to the threshold MDC value for each test. Finally, survey responses were manually tabulated for the reported number of falls and the percentage of positive or negative responses to questionnaire items.

## Results

### Participant Characteristics

**Figure 2.**
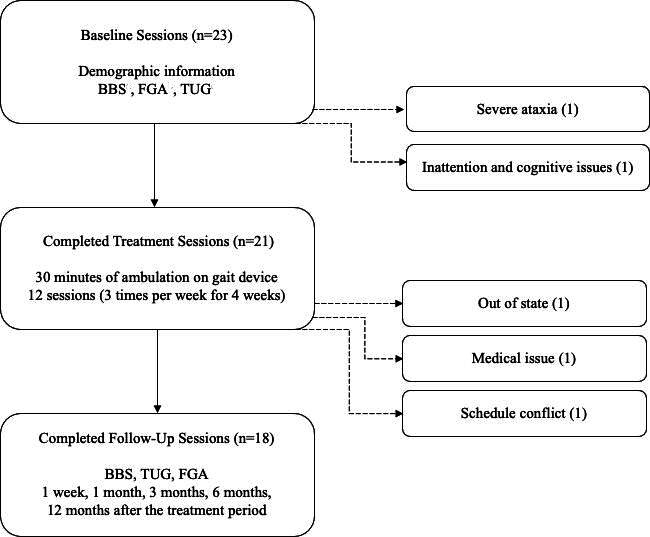
Number of study participants at each stage of the research study. Key study activities are listed within each phase, and reasons for nonparticipation are available to the right of each phase heading. BBS: Berg Balance Scale; FGA: Functional Gait Assessment; TUG: Timed Up and Go.

Figure 2 shows the study activities and the number of participants in each study phase from recruitment through follow-up. Recruitment occurred between July 2018 and September 2018. Treatment occurred between July 2018 and December 2018, and all study-related follow-up was completed in December 2019. The results and analyses of this study include the 18 study participants who completed all assessments through the 12-month follow-up. The demographics of the study participants are shown in Table 1.

**Table 1.** Participant demographics.

ID	Sex	Weight (lbs)	Age (years)	Time since stroke (months)	Side of hemiparesis	AFO^a^	Treatment duration (minutes)
A	M^b^	160	53	24	Left	No	295
B	M	198	55	13	Right	Yes	355
C	F^c^	200	44	14	Left	No	270
D	M	240	51	50	Left	Partial^d^	360
E	M	235	52	130	Right	Yes	360
F	F	134	46	308	Left	No	360
G	M	220	61	22	Left	Yes	290
H	M	190	63	53	Left	Yes	285
I	F	250	58	92	Left	No	360
J	F	222	69	89	Left	No	330
K	F	189	47	28	Right	Partial^d^	215
L	F	172	53	32	Left	No	360
M	F	183	50	80	Left	Yes	210
N	M	134	61	46	Left	Partial^d^	165
O	F	150	54	21	Right	No	280
P	F	180	77	15	Left	No	170
Q	M	175	64	30	Right	Yes	260
R	M	188	62	28	Left	No	295
Total	18 (9 M and 9 F)	Mean (SD) 190 (34)	Mean (SD) 57 (9)	Mean (SD) 60 (70)	5 Right 13 Left	6 Yes 9 No 3 Partial	Mean (SD) 290 (66)

a AFO: Ankle-Foot Orthosis.

b M: male.

c F: female.

d Partial:participantss who used an AFO occasionally at baseline, but did not use during the study.

### Statistical Analysis

Mauchly Test of Sphericity showed significance, indicating a violation of one of the assumptions of a repeated-measures ANOVA for the TUG and BBS (but not the FGA); therefore, Greenhouse-Geisser corrections were applied. Further, TUG did not show normally distributed results; therefore, we used the Friedman test for the TUG (BBS and FGA were normally distributed for all except one time frame). Using repeated-measures ANOVA, our findings showed statistical significance from baseline to each follow-up time frame for BBS (*F*_2.5,42.8_=19.5; *P*<.001) and FGA (*F*_5,85_=18.3; *P*<.001). Friedman test also showed statistical significance from baseline to each follow-up time frame for TUG, (*χ*^2^_5_=33.0; *P*<.001). Table 2 shows the outcome measure means at each assessment session, outcome measure score changes relative to baseline, and associated *P* values. Respective MDC values and fall risk thresholds are provided in the table for reference. Note that the percentage difference calculation for the FGA is the average of each participant’s percent change score.

The relationship between the duration of treatment with the gait device (in min) and balance assessment scores at each posttreatment time period was assessed using Spearman rank-order correlation coefficient. Table 3 shows that statistically significant moderate to strong correlations were found between treatment minutes and the majority of balance scores at each of the 5 follow-up sessions. Statistically significant relationships are indicated in the table below.

**Table 2.** Statistical analysis, from baseline to each posttreatment time frame.

Values	Baseline	1 wk post treatment	1 mo post treatment	3 mo post treatment	6 mo post treatment	12 mo post treatment
BBS^a^ (pts^b^) MDC^c^=2.5 pts [38]; fall risk≤44 pts [37]						
Mean (SD)	42.6 (6.0)	46.7 (4.8)	48.5 (4.8)	49.3 (5.2)	48.8 (5.4)	48.5 (5.8)
Difference	—^d^	4.1	5.9	6.7	6.2	5.9
*P* value	—	.01^e^	<.001^e^	^e^<.001^f^	<.001^e^	.001^e^
TUG^f^ (sec) MDC=−3.2 sec [34]; fall risk≥14 sec [43]						
Mean (SD)	19.7 (8.6)	14.9 (6.1)	14.4 (6.0)	14.4 (5.8)	14.1 (6.6)	14.8 (6.9)
Difference	—	–4.8	−5.3	−5.3	−5.6	−4.9
*P* value	—	<.001^e^	.001^e^	.001^e^	.004^e^	.05^e^
FGA^g^ (pts)						
Mean (SD)	15.1 (5.1)	20.1 (4.6)	20.7 (4.3)	21.7 (4.5)	20.4 (4.5)	18.9 (4.5)
MDC=4.2 pts or 14.1% [46]						
Difference	—	5.0	5.6	6.6	5.3	3.8
Fall risk≤15 pts [45]						
Difference	—	41.4	48.2	56.5	45.4	34.6
*P* value	—	<.001^e^	<.001^e^	<.001^e^	<.001^e^	.005^e^

a BBS: Berg Balance Scale.

b pts: points.

c MDC: minimal detectable change.

d Not applicable.

e Statistically significant, *P*<.05.

f TUG: Timed Up and Go.

g FGA: Functional Gait Assessment.

**Table 3.** Correlation analysis between balance scores at each time period and minutes of treatment.

Outcome and balance measurement	Minutes of treatment	
	Spearman ρ	*P* value
BBS^a^		
BBS 1 wk post treatment	0.574^b^	.01
BBS 1 mo post treatment	0.624^b^	.006
BBS 3 mo post treatment	0.634^b^	.005
BBS 6 mo post treatment	0.442	.07
BBS 12-mo post treatment	0.400	.10
TUG^c^		
TUG 1 wk post treatment	−0.549^b^	.02
TUG 1 mo post treatment	−0.613^b^	.007
TUG 3 mo post treatment	−0.640^b^	.004
TUG 6 mo post treatment	−0.560^b^	.02
TUG 12 mo post treatment	−0.749^b^	<.001
FGA^d^		
FGA 1 wk post treatment	0.118	.64
FGA 1 mo post treatment	−0.036	.89
FGA 3 mo post treatment	0.157	.53
FGA 6 mo post treatment	0.252	.31
FGA 12 mo post treatment	0.485^b^	.04

a BBS: Berg Balance Scale.

b Statistically significant correlation, *P*<.05.

c TUG: Timed Up and Go.

d FGA: Functional Gait Assessment

### Balance Improvement

#### Overview

**Figure 3.**
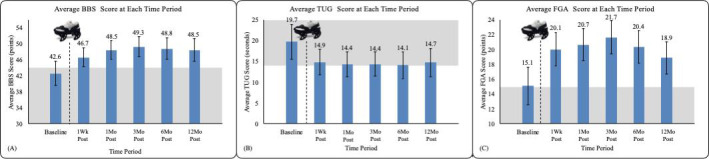
Average outcome measure score across all study participants (n=18) at each time period. (**A**) Average Berg Balance Scale score at each study time period. (**B**) Average Timed Up and Go score at each study time period. (**C**) Average Functional Gait Assessment score at each study time period. Error bars represent 95% CIs, and shading corresponds to each outcome’s fall risk threshold. BBS: Berg Balance Scale; FGA: Functional Gait Assessment; TUG: Timed Up and Go. Post: Posttreatment.

The average score on each balance measure at each time period is shown in Figures 3A-3C, with the respective threshold fall risk range indicated with gray shading for comparison. For the BBS, the average score improved from below the fall risk threshold (ie, high risk for falls) at baseline to above the fall risk threshold (ie, low risk for falls) at each of the 5 follow-ups. For the TUG, the average score at each time period improved by more than 4 seconds and approached the fall risk threshold score of 14 seconds [43]. Finally, the average FGA score of 15.1 approximates the fall threshold score of 15 points [45] at baseline but improves over 3 points beyond the threshold at each posttreatment assessment.

#### Improvement Relative to the Minimal Detectable Change

The MDC determines the smallest amount of change that is not likely due to chance variation in measurement [47]. Therefore, we sought to determine if the magnitude of posttreatment measurement changes (for each participant at each time frame) exceeded this metric. Table 4 shows a comparison to the MDC. Note that some individuals scored highly on the balance measures at baseline (such as participant R, who scored 8.8 s on the TUG, 52 on the BBS, and 26 on the FGA at baseline). Therefore, exceeding an MDC threshold may not have been expected for some participants. Nonetheless, findings show that at least 72% (13/18) of participants exceeded the MDC of BBS, at least 44% (8/18) exceeded the MDC of TUG, and at least 66% (12/18) exceeded the MDC of FGA at every posttreatment time point.

**Table 4.** Improvement relative to the minimal detectable change (✓ indicates improvement beyond minimal detectable change, and — indicates a change less than minimal detectable change).

ID	1 wk post treatment			1 mo post treatment			3 mo post treatment			6 mo post treatment			12 mo post treatment			%^a^
	BBS	TUG	FGA	BBS	TUG	FGA	BBS	TUG	FGA	BBS	TUG	FGA	BBS^b^	TUG^c^	FGA^d^	
A	**✓**	**✓**	**✓**	**✓**	**✓**	**✓**	**✓**	**✓**	**✓**	**✓**	**✓**	**✓**	**✓**	**✓**	**✓**	100
B	**✓**	**✓**	**✓**	**✓**	**✓**	**✓**	**✓**	**✓**	**✓**	**✓**	**✓**	**✓**	**✓**	**✓**	**✓**	100
C	**✓**	**✓**	**✓**	**✓**	**✓**	**✓**	**✓**	**✓**	**✓**	**✓**	**✓**	**✓**	**✓**	—	**✓**	93
D	**✓**	**✓**	**✓**	**✓**	**✓**	**✓**	**✓**	**✓**	—	**✓**	**✓**	**✓**	**✓**	**✓**	**✓**	93
E	**✓**	—	**✓**	**✓**	**✓**	**✓**	**✓**	**✓**	**✓**	**✓**	**✓**	**✓**	**✓**	**✓**	**✓**	93
F	**✓**	**✓**	**✓**	**✓**	**✓**	**✓**	**✓**	**✓**	**✓**	**✓**	—	**✓**	**✓**	—	**✓**	87
G	**✓**	**✓**	**✓**	**✓**	—	**✓**	**✓**	**✓**	**✓**	**✓**	**✓**	**✓**	**✓**	**✓**	—	87
H	**✓**	**✓**	—	**✓**	**✓**	**✓**	**✓**	**✓**	**✓**	**✓**	**✓**	**✓**	**✓**	**✓**	—	87
I	**✓**	—	**✓**	**✓**	**✓**	**✓**	**✓**	**✓**	**✓**	**✓**	**✓**	**✓**	—	**✓**	**✓**	87
J	**✓**	—	**✓**	**✓**	**✓**	**✓**	**✓**	—	**✓**	**✓**	**✓**	**✓**	—	—	**✓**	73
K	—	**✓**	**✓**	—	**✓**	**✓**	—	—	**✓**	**✓**	**✓**	—	**✓**	**✓**	**✓**	67
L	**✓**	—	**✓**	**✓**	—	**✓**	**✓**	—	**✓**	**✓**	—	**✓**	**✓**	—	**✓**	67
M	**✓**	—	**✓**	**✓**	—	**✓**	**✓**	—	**✓**	**✓**	—	**✓**	**✓**	—	**✓**	67
N	—	**✓**	**✓**	—	**✓**	**✓**	—	**✓**	**✓**	**✓**	—	**✓**	—	—	**✓**	60
O	—	**✓**	—	**✓**	**✓**	—	**✓**	**✓**	—	**✓**	**✓**	—	**✓**	—	—	53
P	**✓**	—	**✓**	**✓**	**✓**	**✓**	**✓**	—	—	—	**✓**	**✓**	—	—	—	53
Q	—	—	**✓**	—	—	**✓**	—	—	**✓**	—	—	**✓**	**✓**	—	—	33
R	—	—	—	—	—	—	—	—	—	—	—	—	—	—	—	0
%^e^	72.2	55.6	83.3	77.8	72.2	88.9	77.8	61.1	77.8	83.3	66.7	83.3	72.2	44.4	66.7	

a Percentage of time periods each individual participant exceeded the MDC.

b BBS: Berg Balance Scale.

c TUG: Timed Up and Go.

d FGA: Functional Gait Assessment.

e Percentage of participants that improved greater than the MDC at each time period.

### Fall Risk Classification

**Figure 4.**
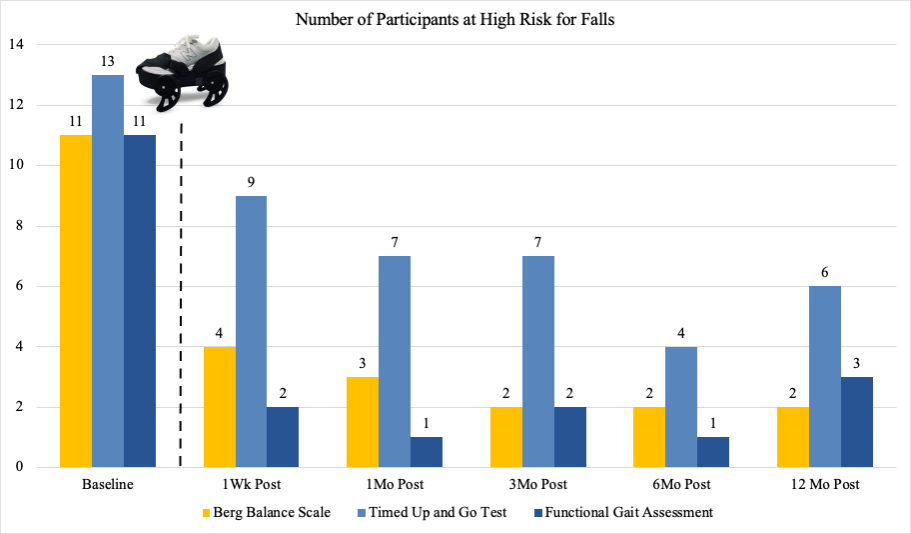
Number of participants at high risk for falls, as determined by the Berg Balance Scale, the Timed Up and Go test, and the Functional Gait Assessment, at each study time period. The Functional Gait Assessment uses the Parkinson disease fall risk threshold. Post: posttreatment.

The number of participants classified as high fall risk by each assessment’s criteria is shown in Figure 4. For the BBS, 11 (61%) participants were categorized as a high fall risk at baseline [38], but only 2-4 participants remained at high fall risk after treatment. Thirteen (72%) participants scored as high fall risk using TUG criteria at baseline, and this number was reduced to 4-9 participants at each posttreatment time period. Similarly, while 11 (61%) participants scored at high fall risk at baseline on the FGA, only 1-3 participants remained at high risk for falls after treatment with the gait device. Of the 15 participants who were identified as being at risk for falls at baseline, 15 out of 15 (100%) reduced their risk for falls on at least one outcome (during at least one posttreatment measurement).

### Outcome Measure Agreement

**Figure 5.**
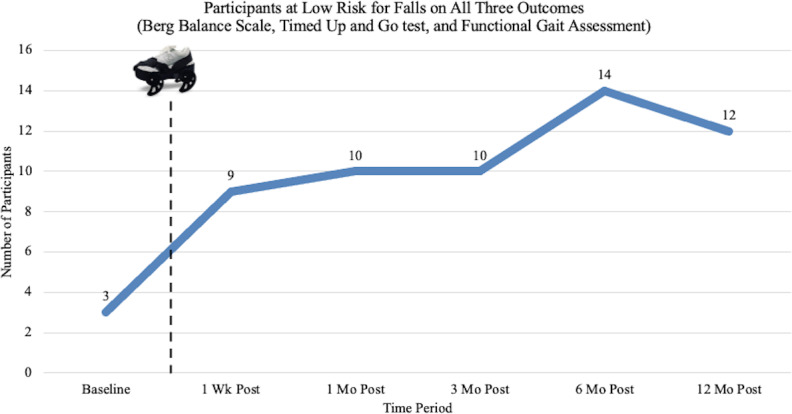
Number of participants at low risk for fall on all 3 outcome measures (the Berg Balance Scale, the Timed Up and Go test, and the Functional Gait Assessment), at each study time period. Post: posttreatment.

Fall risk classifications on all 3 functional balance assessments are combined into Figure 5. Findings show a sustained and progressive increase in the number of participants who scored as low risk for falls on all measures through the 6-month follow-up. At 12 months post treatment, the number of participants at low risk for falls remains substantially above baseline.

### Questionnaire Results

**Figure 6.**
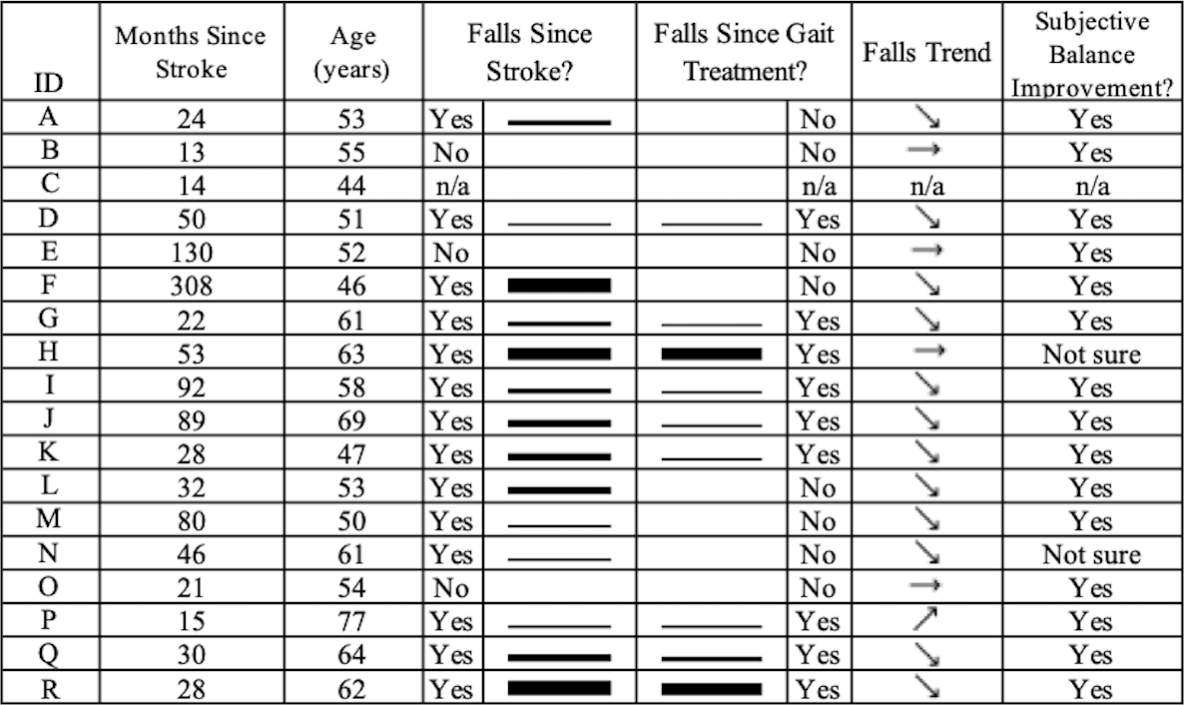
Questionnaire results. Sparkline thickness correlates with the number of sustained falls, with thicker lines indicating a higher number of falls. Months since stroke and age are provided for further context of the results. A downward-pointing arrow represents a trend toward fewer falls, an upward-pointing arrow represents a trend toward more falls, and a horizontal arrow represents no change in the fall trend.

Participants were questioned on the frequency of falls since their stroke and in the 12 months after being treated with the gait device. Questionnaire answer responses for the number of falls included the following ranges: (1) no falls, (2) 1‐5 falls, (3) 5‐10 falls, and (4)>10 falls. In addition, participants were asked if the gait device improved their balance. Questionnaire results are provided in Figure 6. Note that since the number of falls was provided in a numerical range and the duration of time between fall reference durations is variable (ie, the duration of time since each participant’s stroke and the duration of time since their treatment with the gait device), sparklines are used as a visual representation of the data trends. In addition, 1 participant did not complete the questionnaire (indicated by “n/a” in the table).

## Discussion

### Principal Findings

In this study, we measured participants’ functional balance using the BBS, the TUG test, and the FGA before and several times after treatment with the gait device. Results reveal statistically significant improvements on each outcome at all 5 posttreatment time frames (*P*<.05) and a substantial reduction in the number of individuals categorized as high risk for falls by each of these outcome measures.

In addition to improved functional balance scores, the retention of balance improvement is a key finding of this study. Twelve months posttreatment, scores on each functional outcome remained significantly improved compared with baseline measurements. Moreover, using thresholds reported in the literature to classify fall risk, 12 out of 18 (67%) participants scored as low risk for falls on each of the three outcomes 12 months post treatment, an improvement from 3 out of 18 (17%) before treatment. A 2010 systematic review by Lubetzky-Vilnai and Kartin [48] analyzed the effect of balance training on balance performance for poststroke populations of varying chronicity. In comparison to the eight cited studies focused on the chronic stroke population with a comparable outcome measure [49-56], the majority of studies (5/8, 63%) report an immediate post treatment BBS improvement of lesser magnitude than the present study [49,51,52,55,56], although most of these studies demonstrated a more robust study design. Three studies reported greater improvement on the BBS compared with our study; however, these studies had longer or more intense treatment regimens [50,53,54]. In addition, none of these studies demonstrate retention of the reported improvements beyond 3 months after treatment.

The specific factors determining the retention of balance-related treatment effects post stroke lack substantial discussion in rehabilitation literature. However, research suggests that achieving improved performance of an affected limb or improved mobility levels after stroke may further enable improvement-sustaining activity participation [57-59]. In our study, a notable improvement in functional balance, in combination with other functional parameters targeted by the gait device (ie, asymmetry and gait speed [24,27,29]) could mitigate some of the barriers that limit poststroke mobility participation, subsequently sustaining the treatment effects. Future studies could clarify the exact mechanisms that translate to higher balance measure scores, and especially the retention of these effects, after treatment with the gait device.

While functional balance measures are routinely used in research and clinical practice to reflect risk for falls and fall risk reduction following treatment, it is important to correlate results on these measures with real-world fall rates. In this study, a posttreatment questionnaire regarding the participant’s fall history (from the date of their stroke event until the start of the clinical trial and in the 12 months following gait device treatment) was used to capture this information. Fourteen of 17 (82%) participants classified themselves as experiencing one or more falls before the start of the clinical trial. While this percentage appears high compared with rehabilitation literature [2], it is in line with studies reporting that those most likely to fall have moderate function (ie, impaired ability to maintain balance, yet still reasonably mobile) as found in our study population [60]. In the year following treatment with the gait device, the number of participants experiencing falls was reduced to 9 of 17 (53%), and some of the remaining 8 participants appeared to reduce their fall frequency. While the relative time duration of the pre- and posttreatment periods is not consistent for most participants (months since stroke is included in Figure 6 to provide context), a beneficial trend in real-world falls likely occurred. Importantly, the 5 oldest study participants (ages 62‐77 years) remained fallers posttreatment, and 67% of those who fell after the treatment period were older than 60 years. These results highlight the well-known additional impact of aging on fall risk, including for those with stroke [61]. Moreover, the continued occurrence of falls despite improved balance function highlights the complexity surrounding falls management and the need for comprehensive assessments and treatments, in addition to gait and balance treatment, for individuals post stroke.

### Limitations

Our study has several limitations. First, without a control group, we cannot exclude the possibility of confounding variables influencing our results. This limitation reduces our ability to draw definitive causal conclusions about the effectiveness of the intervention and limits generalizability to a broader poststroke populations or circumstances. Therefore, these results should be regarded as preliminary and in need of further confirmation with larger, controlled studies. A second limitation is attaining a falls history using a self-report questionnaire, which may be unreliable and subject to bias. In addition, the use of categorical measures for fall rates limits direct pre-post fall comparisons. These results could be strengthened by monitoring fall occurrence rates before, during, and after the treatment period. In addition, as the therapist assessors also oversaw the treatment, examiner bias is possible, and testing effects can occur with repeated outcome measurement.

Further, as noted in our inclusion criteria, the clinical trial participants did not receive additional physical therapy treatment from the clinical trial physical therapists or external physical therapists during the treatment period. However, given the extended duration of our study (ie, greater than 1 year), we did not exclude participants for obtaining additional therapy after the 1-week follow-up assessment was completed. Six of the 18 participants (B, D, H, M, Q, and R) did receive some form of therapy treatment between the 1-week and 12-month follow-up. Of these 6 participants, 3 had minimal treatment (6 or fewer therapy sessions), and of the remaining 3 participants, 2 received services focusing on upper extremity function (with some full-body therapy included). Importantly, two-thirds of our participants (12/18 participants) did not receive any additional physical therapy treatment throughout the study duration. Also, reviewing our results without the data from these 6 individuals yields largely unchanged findings (and even stronger trends in the percentages of participants exceeding MDC values). These results are shown in Multimedia Appendix 1.

Finally, in our results, we compare the participants’ improvement posttreatment to each outcome measure’s corresponding MDC value. While MDC provides a threshold to exceed the measurement error of each test, it does not provide context on the meaningfulness of the improvement (ie, minimal clinically important difference [MCID]), arguably a more important attribute post treatment. Unfortunately, MCID thresholds were not available for the selected outcomes in the population of chronic stroke. Several populations have been studied to obtain MCID values for these outcomes, including multiple sclerosis and the BBS [62] (MCID of 3 points compared with our used MDC value of 2.5 points), community-dwelling older adults and individuals with vestibular disorders for the FGA [63,64] (MCID of 4 points compared our used MDC value of 4.2 points or 14.1%), and patients undergoing lumbar surgery for the TUG test [65] (MCID of −3.4 s compared with our used MDC value of −3.2 s). While not directly generalizable, the similarities of these values to the MDC values for the chronic stroke population may suggest the meaningfulness of the improvement noted in this study.

### Conclusions

The results of this study indicate that treatment with the gait device has the potential to result in long-term functional balance improvement for individuals with chronic stroke. Our findings reveal significantly improved scores on 3 validated balance measures, with improvements retained one year after treatment. In addition, score changes reflect a reduced risk of falls in the year following treatment with the gait device. Larger, controlled studies could confirm these results and verify the reduction in fall rates through pre- and posttreatment monitoring.

## Supplementary material

10.2196/67297 Multimedia Appendix 1 Improvement relative to the minimal detectable change with participants who received some additional therapy during posttreatment follow-ups removed from analysis (the plus symbol indicates improvement beyond MDC, and the dot symbol indicates a change less than MDC). With individuals who received some additional therapy during posttreatment follow-ups removed from analysis, the percentage of participants who improved greater than the MDC at each time period increased for 12 of the 15 measurements compared with Table 4.

## Abbreviations

BBS: Berg Balance Scale
FGA: Functional Gait Assessment
HIPAA: Health Insurance Portability and Accountability Act
IRB: institutional review board
MCID: minimal clinically important difference
MDC: minimal detectable change
TUG: Timed Up and Go
WCG: Western-Copernicus Group

## References

[1] Sackley C, Brittle N, Patel S (2008). The prevalence of joint contractures, pressure sores, painful shoulder, other pain, falls, and depression in the year after a severely disabling stroke. Stroke.

[2] Weerdesteyn V, de Niet M, van Duijnhoven HJR, Geurts ACH (2008). Falls in individuals with stroke. J Rehabil Res Dev.

[3] Forster A, Young J (1995). Incidence and consequences of falls due to stroke: a systematic inquiry. BMJ.

[4] Batchelor FA, Mackintosh SF, Said CM, Hill KD (2012). Falls after stroke. Int J Stroke.

[5] Kapral MK, Fang J, Alibhai SMH (2017). Risk of fractures after stroke: results from the Ontario stroke registry. Neurology (ECronicon).

[6] Field MJ, Gebruers N, Shanmuga Sundaram T, Nicholson S, Mead G (2013). Physical activity after stroke: a systematic review and meta-analysis. ISRN Stroke.

[7] Jørgensen L, Engstad T, Jacobsen BK (2002). Higher incidence of falls in long-term stroke survivors than in population controls. Stroke.

[8] Schmid AA, Van Puymbroeck M, Altenburger PA (2012). Balance and balance self-efficacy are associated with activity and participation after stroke: a cross-sectional study in people with chronic stroke. Arch Phys Med Rehabil.

[9] Hong E (2015). Health-related quality of life of community-dwelling stroke survivors: a comparison of fallers and non-fallers. J Phys Ther Sci.

[10] Lamb SE, Ferrucci L, Volapto S, Fried LP, Guralnik JM (2003). Risk factors for falling in home-dwelling older women with stroke. Stroke.

[11] Verheyden G, Weerdesteyn V, Pickering RM (2013). Interventions for preventing falls in people after stroke. Cochrane Database Syst Rev.

[12] Batchelor F, Hill K, Mackintosh S, Said C (2010). What works in falls prevention after stroke?. Stroke.

[13] Denissen S, Staring W, Kunkel D (2019). Interventions for preventing falls in people after stroke. Cochrane Database Syst Rev.

[14] Arienti C, Lazzarini SG, Pollock A, Negrini S (2019). Rehabilitation interventions for improving balance following stroke: an overview of systematic reviews. PLoS ONE.

[15] Lackland DT, Roccella EJ, Deutsch AF (2014). Factors influencing the decline in stroke mortality: a statement from the American Heart Association/American Stroke Association. Stroke.

[16] Reed KB, Handzic I, University of South Florida (2016). Gait altering shoes.

[17] Patterson KK, Parafianowicz I, Danells CJ (2008). Gait asymmetry in community-ambulating stroke survivors. Arch Phys Med Rehabil.

[18] An CM, Son YL, Park YH, Moon SJ (2017). Relationship between dynamic balance and spatiotemporal gait symmetry in hemiplegic patients with chronic stroke. Hong Kong Physiother J.

[19] Handz̆ić I, Reed KB (2014). Kinetic shapes: analysis, verification, and applications. J Mech Des N Y.

[20] Olney SJ, Richards C (1996). Hemiparetic gait following stroke. Part I: characteristics. Gait Posture.

[21] Dos Anjos S, Morris D, Taub E (2020). Constraint-induced movement therapy for lower extremity function: describing the LE-CIMT protocol. Phys Ther.

[22] Reisman DS, McLean H, Keller J, Danks KA, Bastian AJ (2013). Repeated split-belt treadmill training improves poststroke step length asymmetry. Neurorehabil Neural Repair.

[23] Handzic I, Barno EM, Vasudevan EV, Reed KB (2011). Design and pilot study of a gait enhancing mobile shoe. Paladyn.

[24] Kim SH, Huizenga DE, Handzic I (2019). Relearning functional and symmetric walking after stroke using a wearable device: a feasibility study. J Neuroeng Rehabil.

[25] Handzic I, Vasudevan E, Reed KB (2012). Developing a gait enhancing mobile shoe to alter over-ground walking coordination. IEEE Int Conf Robot Autom.

[26] Kim SH HI H, Edgeworth R, Lazinski M, Ramakrishnan T, Reed KB Inital results of the gait enhancing mobile shoe on individuals with stroke.

[27] Huizenga D, Rashford L, Darcy B (2021). Wearable gait device for stroke gait rehabilitation at home. Top Stroke Rehabil.

[28] Darcy B, Rashford L, Shultz ST (2023). Gait device treatment using telehealth for individuals with stroke during the COVID-19 pandemic: nonrandomized pilot feasibility study. JMIR Form Res.

[29] Darcy B, Rashford L, Tsai NT, Huizenga D, Reed KB, Bamberg SJM (2023). One-year retention of gait speed improvement in stroke survivors after treatment with a wearable home-use gait device. Front Neurol.

[30] Mancini M, Horak FB (2010). The relevance of clinical balance assessment tools to differentiate balance deficits. Eur J Phys Rehabil Med.

[31] Bower K, Thilarajah S, Pua YH (2019). Dynamic balance and instrumented gait variables are independent predictors of falls following stroke. J Neuroeng Rehabil.

[32] Sibley KM, Straus SE, Inness EL, Salbach NM, Jaglal SB (2011). Balance assessment practices and use of standardized balance measures among Ontario physical therapists. Phys Ther.

[33] Bushnell C, Bettger JP, Cockroft KM (2015). Chronic Stroke Outcome Measures for Motor Function Intervention Trials: Expert Panel Recommendations. Circ Cardiovasc Qual Outcomes.

[34] Alghadir AH, Al-Eisa ES, Anwer S, Sarkar B (2018). Reliability, validity, and responsiveness of three scales for measuring balance in patients with chronic stroke. BMC Neurol.

[35] Moore JL, Potter K, Blankshain K, Kaplan SL, OʼDwyer LC, Sullivan JE (2018). A core set of outcome measures for adults with neurologic conditions undergoing rehabilitation: A CLINICAL PRACTICE GUIDELINE. J Neurol Phys Ther.

[36] Berg KO, Wood-Dauphinee SL, Williams JI, Maki B (1992). Measuring balance in the elderly: validation of an instrument. Can J Public Health.

[37] Simpson LA, Miller WC, Eng JJ (2011). Effect of stroke on fall rate, location and predictors: a prospective comparison of older adults with and without stroke. PLoS One.

[38] Liston RA, Brouwer BJ (1996). Reliability and validity of measures obtained from stroke patients using the Balance Master. Arch Phys Med Rehabil.

[39] Flansbjer UB, Blom J, Brogårdh C (2012). The reproducibility of Berg Balance Scale and the Single-leg Stance in chronic stroke and the relationship between the two tests. PM R.

[40] Hiengkaew V, Jitaree K, Chaiyawat P (2012). Minimal detectable changes of the Berg Balance Scale, Fugl-Meyer Assessment Scale, Timed “Up & Go” Test, gait speeds, and 2-minute walk test in individuals with chronic stroke with different degrees of ankle plantarflexor tone. Arch Phys Med Rehabil.

[41] Podsiadlo D, Richardson S (1991). The timed “Up & Go”: a test of basic functional mobility for frail elderly persons. J Am Geriatr Soc.

[42] Ng SS, Hui-Chan CW (2005). The timed up & go test: its reliability and association with lower-limb impairments and locomotor capacities in people with chronic stroke. Arch Phys Med Rehabil.

[43] Andersson AG, Kamwendo K, Seiger A, Appelros P (2006). How to identify potential fallers in a stroke unit: validity indexes of 4 test methods. J Rehabil Med.

[44] Wrisley DM, Marchetti GF, Kuharsky DK, Whitney SL (2004). Reliability, internal consistency, and validity of data obtained with the functional gait assessment. Phys Ther.

[45] Leddy AL, Crowner BE, Earhart GM (2011). Functional gait assessment and balance evaluation system test: reliability, validity, sensitivity, and specificity for identifying individuals with Parkinson disease who fall. Phys Ther.

[46] Lin JH, Hsu MJ, Hsu HW, Wu HC, Hsieh CL (2010). Psychometric comparisons of 3 functional ambulation measures for patients with stroke. Stroke.

[47] Haley SM, Fragala-Pinkham MA (2006). Interpreting change scores of tests and measures used in physical therapy. Phys Ther.

[48] Lubetzky-Vilnai A, Kartin D (2010). The effect of balance training on balance performance in individuals poststroke: a systematic review. J Neurol Phys Ther.

[49] Olawale OA, Ogunmakin OS (2006). The effect of exercise training on balance in adult patients with post-stroke hemiplegia. Int J Ther Rehabil.

[50] Fritz SL, Pittman AL, Robinson AC, Orton SC, Rivers ED (2007). An intense intervention for improving gait, balance, and mobility for individuals with chronic stroke: a pilot study. J Neurol Phys Ther.

[51] Yen CL, Wang RY, Liao KK, Huang CC, Yang YR (2008). Gait training induced change in corticomotor excitability in patients with chronic stroke. Neurorehabil Neural Repair.

[52] Leroux A, Pinet H, Nadeau S (2006). Task-oriented intervention in chronic stroke: changes in clinical and laboratory measures of balance and mobility. Am J Phys Med Rehabil.

[53] Macko RF, Benvenuti F, Stanhope S (2008). Adaptive physical activity improves mobility function and quality of life in chronic hemiparesis. J Rehabil Res Dev.

[54] Michael K, Goldberg AP, Treuth MS, Beans J, Normandt P, Macko RF (2009). Progressive adaptive physical activity in stroke improves balance, gait, and fitness: preliminary results. Top Stroke Rehabil.

[55] Stuart M, Benvenuti F, Macko R (2009). Community-based adaptive physical activity program for chronic stroke: feasibility, safety, and efficacy of the Empoli model. Neurorehabil Neural Repair.

[56] Huijbregts MPJ, McEwen S, Taylor D (2009). Exploring the feasibility and efficacy of a telehealth stroke self-management programme: a pilot study. Physiother Can.

[57] Wolf SL, Winstein CJ, Miller JP (2008). Retention of upper limb function in stroke survivors who have received constraint-induced movement therapy: the EXCITE randomised trial. Lancet Neurol.

[58] Wang C, Winstein C, D’Argenio DZ, Schweighofer N (2020). The efficiency, efficacy, and retention of task practice in chronic stroke. Neurorehabil Neural Repair.

[59] Madhavan S, Lim H, Sivaramakrishnan A, Iyer P (2019). Effects of high intensity speed-based treadmill training on ambulatory function in people with chronic stroke: A preliminary study with long-term follow-up. Sci Rep.

[60] Wei WE, De Silva DA, Chang HM (2019). Post-stroke patients with moderate function have the greatest risk of falls: a national cohort study. BMC Geriatr.

[61] Ugur C, Gücüyener D, Uzuner N, Ozkan S, Ozdemir G (2000). Characteristics of falling in patients with stroke. J Neurol Neurosurg Psychiatry.

[62] Gervasoni E, Jonsdottir J, Montesano A, Cattaneo D (2017). Minimal clinically important difference of Berg Balance Scale in people with multiple sclerosis. Arch Phys Med Rehabil.

[63] Beninato M, Fernandes A, Plummer LS (2014). Minimal clinically important difference of the functional gait assessment in older adults. Phys Ther.

[64] Wellons RD, Duhe SE, MacDowell SG, Hodge A, Oxborough S, Levitzky EE (2022). Estimating the minimal clinically important difference for balance and gait outcome measures in individuals with vestibular disorders. J Vestib Res.

[65] Gautschi OP, Stienen MN, Corniola MV (2017). Assessment of the minimum clinically important difference in the Timed Up and Go test after surgery for lumbar degenerative disc disease. Neurosurgery.

