# Supplementary material for: Balance Improvement and Fall Risk Reduction in Stroke Survivors After Treatment With a Wearable Home-Use Gait Device: Single-Arm Longitudinal Study With 1-Year Follow-Up
Source: JMIR Form Res. 2025 Aug 15;9:e67297. doi: 10.2196/67297 (PMC12356525; doi:10.2196/67297)
Supplement: Multimedia Appendix 1 [file formative-v9-e67297-s001.docx]

Multimedia Appendix 1. Improvement relative to the minimal detectable change (MDC) with participants who received some additional therapy during post-treatment follow-ups removed from analysis (the plus symbol indicates improvement beyond MDC, and the dot symbol indicates a change less than MDC). With individuals who received some additional therapy during post-treatment follow-ups removed from analysis, the percentage of participants who improved greater than the MDC at each time period is increased for 12 of the 15 measurements compared to Table 4.

| ID | **1 Wk Post** | | | | **1 Mn Post** | | | **3 Mn Post** | | | **6Mn Post** | | | **12 Mn Post** | | | %^b^ |
| --- | --- | --- | --- | --- | --- | --- | --- | --- | --- | --- | --- | --- | --- | --- | --- | --- | --- |
|  | BBS | TUG | | FGA | BBS | TUG | FGA | BBS | TUG | FGA | BBS | TUG | FGA | BBS | TUG | FGA |  |
| A | **+** | **+** | **+** | | **+** | **+** | **+** | **+** | **+** | **+** | **+** | **+** | **+** | **+** | **+** | **+** | 100 |
| C | **+** | **+** | **+** | | **+** | **+** | **+** | **+** | **+** | **+** | **+** | **+** | **+** | **+** | **•** | **+** | 93 |
| E | **+** | **•** | **+** | | **+** | **+** | **+** | **+** | **+** | **+** | **+** | **+** | **+** | **+** | **+** | **+** | 93 |
| F | **+** | **+** | **+** | | **+** | **+** | **+** | **+** | **+** | **+** | **+** | **•** | **+** | **+** | **•** | **+** | 87 |
| G | **+** | **+** | **+** | | **+** | **•** | **+** | **+** | **+** | **+** | **+** | **+** | **+** | **+** | **+** | **•** | 87 |
| I | **+** | **•** | **+** | | **+** | **+** | **+** | **+** | **+** | **+** | **+** | **+** | **+** | **•** | **+** | **+** | 87 |
| J | **+** | **•** | **+** | | **+** | **+** | **+** | **+** | **•** | **+** | **+** | **+** | **+** | **•** | **•** | **+** | 73 |
| K | **•** | **+** | **+** | | **•** | **+** | **+** | **•** | **•** | **+** | **+** | **+** | **•** | **+** | **+** | **+** | 67 |
| L | **+** | **•** | **+** | | **+** | **•** | **+** | **+** | **•** | **+** | **+** | **•** | **+** | **+** | **•** | **+** | 67 |
| N | **•** | **+** | **+** | | **•** | **+** | **+** | **•** | **+** | **+** | **+** | **•** | **+** | **•** | **•** | **+** | 60 |
| O | **•** | **+** | **•** | | **+** | **+** | **•** | **+** | **+** | **•** | **+** | **+** | **•** | **+** | **•** | **•** | 53 |
| P | **+** | **•** | **+** | | **+** | **+** | **+** | **+** | **•** | **•** | **•** | **+** | **+** | **•** | **•** | **•** | 53 |
| %^a^ | 75.0 | 58.3 | 91.7 | | 83.3 | 83.3 | 91.7 | 83.3 | 66.7 | 83.3 | 91.7 | 75.0 | 83.3 | 66.7 | 41.7 | 75.0 |  |

^a^Percentage of participants that improved greater than the MDC at each time period

^b^Percentage of time periods each individual participant exceeded the MDC
